# *Gymnema lactiferum*: A Review of Its Traditional Applications, Phytochemical Constituents, and Biological Properties

**DOI:** 10.1002/fsn3.4530

**Published:** 2024-10-16

**Authors:** D. M. K. P. Weerasinghe, L. Brough, D. W. Everett, A. Rashidinejad

**Affiliations:** ^1^ School of Food Technology and Natural Sciences Massey University Palmerston North New Zealand; ^2^ Riddet Institute Massey University Palmerston North New Zealand

**Keywords:** anti‐hyperglycemic activity, antioxidant activity, bioactive compounds, *Gymnema lactiferum*, gymnemic acid

## Abstract

Humanity has a longstanding reliance on natural plants for medicinal purposes, and *Gymnema lactiferum* (*G*. *lactiferum*) has emerged as a medicinal plant with deep‐rooted traditional usage. Throughout history, this plant has been an integral part of traditional medical systems, demonstrating diverse therapeutic effects. Notably, among these effects is its ability to decrease blood glucose concentration in diabetic patients, impart cooling effects, serve as an anabolic and rehydrating agent, stimulate spermiogenesis, and exhibit wormicidal properties. Furthermore, *G*. *lactiferum* has been used in treating conditions such as hemorrhoids cancers, anorexia, and as a cardiac stimulant. The primary objective of this review is to comprehensively gather and critically assess research findings regarding the medicinal properties of *G*. *lactiferum*, specifically emphasizing the bioactive compounds responsible for these properties. Previous studies have documented the presence of various phytochemicals in *G*. *lactiferum*, which are associated with some biological activities, including antioxidative, anti‐hyperglycemic, cholesterol‐regulating, and anti‐inflammatory properties. Additionally, this review explores potential future applications for this plant. Beyond its medicinal significance, extracts derived from *G*. *lactiferum* demonstrate promise for future nutritional applications. This review highlights the potential use of *G*. *lactiferum* as an herbal medicine by critically assessing research on its medicinal value.

## Graphical abstract

*Gymnema lactiferum* (*G*. *lactiferum*) is a traditionally used medicinal plant known for its diverse therapeutic effects. It can lower blood glucose levels, provide cooling effects, stimulate spermiogenesis, and exhibit wormicidal properties. It is also used to treat conditions like hemorrhoids, cancers, anorexia, and as a cardiac stimulant. Studies have identified various phytochemicals in *G*. *lactiferum* with antioxidative, anti‐hyperglycemic, cholesterol‐regulating, and anti‐inflammatory properties.

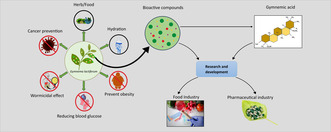

## 1 Introduction

Humanity's enduring reliance on the healing properties of plants has established a long standing tradition of herbal medicine. This dependence on botanicals for healing is fueled by factors such as population growth, limited drug accessibility, adverse side effects associated with synthetic drugs, and escalating healthcare costs. The global demand for medicinal plants has consequently surged, especially for their efficacy in addressing non‐communicable diseases such as diabetes, hypertension, and heart disease.

In Southeast Asia, herbal medicines occupy a prominent position within traditional health systems, including Ayurveda, Siddha, and traditional Chinese medicine (Ansari 2021), underscoring their socio‐cultural significance. An increasing interest in natural remedies is driven by minimal side effects and cost‐effectiveness, reflecting society's inclination toward alternative treatments.

Researchers have extensively explored plant secondary metabolites, unveiling diverse biological activities. In particular, polyphenols, which are abundant in plants, exhibit potent antioxidant properties having value in combating diseases arising from oxidative stress. Antioxidants present in leafy vegetables, such as flavonoids and catechins, play a pivotal role in neutralizing the detrimental effects of free radicals, thus helping to manage chronic conditions such as cancer, anorexia, hemorrhoids (Ramzan et al. 2019) and diabetes (Bandara 2008, 2014; Bandara, Begum, et al. 2010; Bandara, Ekanayake, et al. 2010; Bandara et al. 2009; Gunathilake, Ranaweera, Rupasinghe, and Rathnayaka 2018). Additionally, these compounds serve as defensive mechanisms against pathogens and herbivores while acting as crucial signaling molecules (Deyalage et al. 2021; Gunathilake, Ranaweera, and Rupasinghe 2018a, 2018b; Gunathilake and Ranaweera 2016; Kathirgamanathar, Geethika, Premakumar, Balasubramaniam, et al. 2013).

*Gymnema lactiferum* (*L*.) *R*. *Br*. *ex Schult*. grows in tropical and subtropical regions and is closely related to *Gymnema sylvestre* (*G*. *sylvestre*) within the Apocynaceae flowering plant family. This herbal plant has an extensive history of therapeutic use, particularly in traditional polyherbal formulations for diabetes and other ailments, and has reported antioxidative (Gunathilake, Ranaweera, Rupasinghe, and Rathnayaka 2018, 2016), anti‐inflammatory (Gunathilake, Ranaweera, and Rupasinghe 2018d), anti‐hyperglycemic and blood cholesterol‐controlling properties (Alothman, Bhat, and Karim 2009; Bandara 2008, 2014; Bandara, Begum, et al. 2010; Bandara, Ekanayake, et al. 2010; Bandara et al. 2009). Despite being used as a home remedy for diabetes in Sri Lanka, particularly in the Jaffna area, there is limited published information on its purported health benefits, thus hindering a comprehensive validation of the health effects (Bandara et al. 2009; Gunathilake, Ranaweera, and Rupasinghe 2018a, 2018c, 2018d).

## 2 Nomenclature and Botanical Aspects

The *Gymnema* genus encompasses around 40 diverse species distributed from Western Africa to Australia. Among these species are *G*. *acuminatum* (Roxb.) wall, *G*. *aurantiacum*, *G*. *elegans* W and A, *G*. *balsamicum*, *G*. *tingens* W and A, *G*. *lactiferum*, *G*. *latifolium*, *G*. *sylvestre* R. Br., *G*. *montanum* Hook. F., *G*. *yunnanense*, *G*. *inodorum*, and *G*. *spartum* (Keshavamurthy and Yoganarasimhan 1990).

*Gymnema lactiferum* (*G*. *lactiferum*) is commonly known as the Ceylon cow tree or Ceylon cow plant and referred to as Kurighghan, Muwa Kiri Wel, or Masbedda in Sinhalese (Tennent 1859; Wasana et al. 2021). KiriAguna has been associated with *G*. *lactiferum* in the literature, but more correctly should be ascribed to *G*. *sylvestre*, a closely related variety of *G*. *lactiferum* (Lindley 1853). Gurmar booti in Pakistan (Ramzan et al. 2019), Kurintai in Tamil, and Ksirakakoli in Sanskrit (Dassanayake and Fosberg 1980) all belong to the family *Apocynaceae* and is also known as the dogbane family in some taxa due to its use as a dog poison (Endress and Bruyns 2000). This family of plants primarily thrives in tropical and subtropical latitudes with a few species also found in temperate zones encompassing approximately 400 genera of flowering plants. *G*. *lactiferum* falls under the subfamily *Asclepiadaceae*, commonly referred to as the milkweed family (Saneja et al. 2010). *G*. *lactiferum* is referred to as *Asclepias lactifera* in some documents (Linnaeus 1797). *G*. *lactiferum* has also been associated with the following botanical names: *Asclepias lactifera* L., *G*. *malayanum* Griff., *G*. *nitens* Blume, *G*. *zeylanicum* Decne., *Marsdenia lactifera* (L.) I.M. Turner, *Marsdenia lactifera* var. *thwaitesii* (Hook. f.) I.M. Turner, *G*. *lactiferum* var. khasianum Hook. f., *G*. *lactiferum* var. nitens (Blume) Hook. f., *G*. *lactiferum* var. thwaitesii Hook. f., and *G*. *lactiferum* var. walkeri Hook. f. (IAAM 2008; Pandi 2023).

**FIGURE 1:**
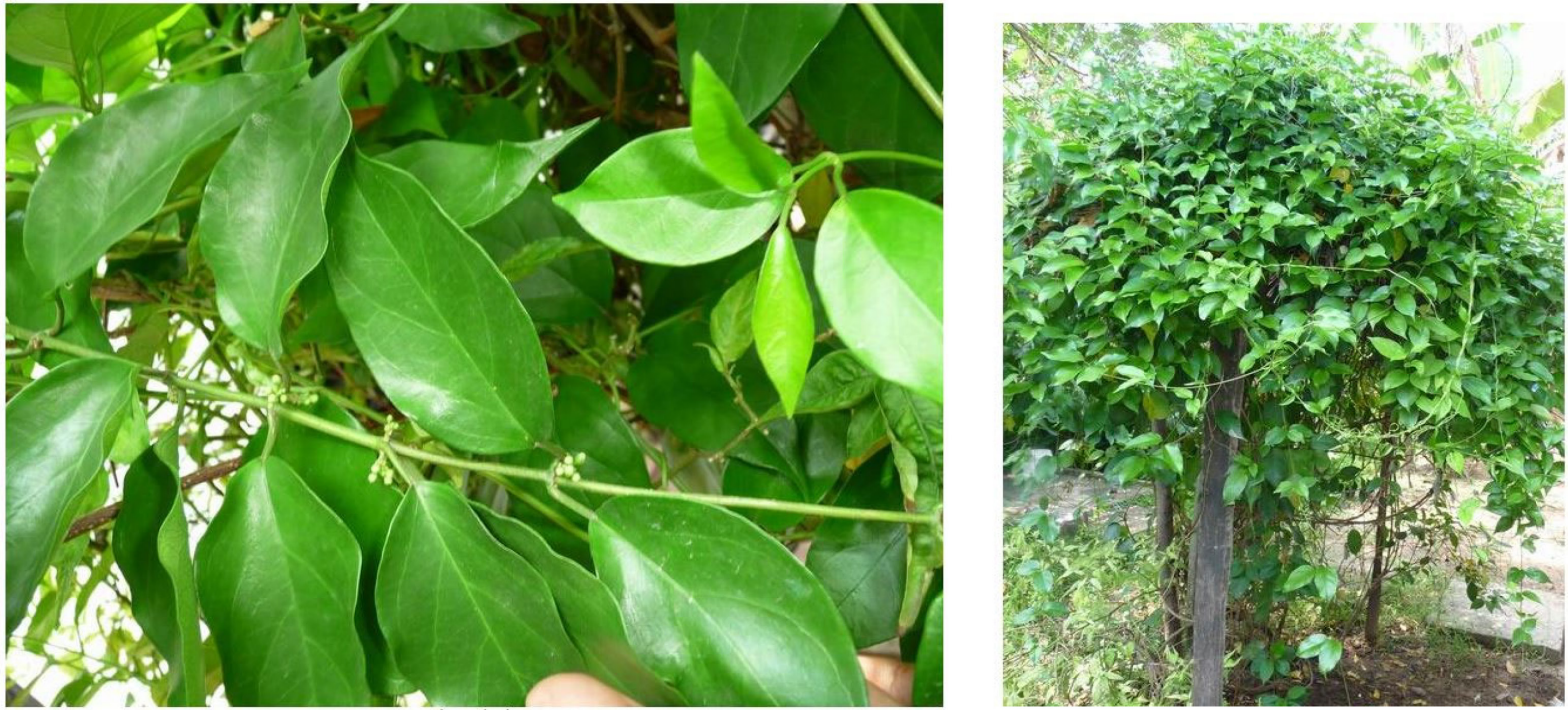
Leaves (left) and the growth behavior (right) of *Gymnema lactiferum*.

Most plants within the *Apocynaceae* family are twining shrubby climbers that exude milky latex, and many of these plants possess medicinal properties (Mahmood et al. 2011). *G*. *lactiferum* also shares this characteristic as a climbing, woody perennial shrub, found in India, Sri Lanka, Indonesia, and some parts of Malaysia. In Sri Lanka, it is primarily found in the Wet zone, although some reports indicate a presence in the evergreen to deciduous forests of the Dry zone (Ramzan et al. 2019). The nomenclature and botanical classification of *G*. *lactiferum* is presented in Table 1. The appearance of *G*. *lactiferum* leaves and the growth pattern are shown in Figure 1.

**TABLE 1:** Taxonomy of *Gymnema lactiferum*.

Kingdom	*Plantae*
Phylum	*Magnoliophyta*
Class	*Magnoliatae*
Order	*Gentianales*
Family	*Apocynaceae* (Subfamily: *Asclepiadaceae*)
Genus	*Gymnema*
Species	*Gymnema lactiferum*

These twining woody plants have puberulous to tomentose coverings. The petiole length ranges from 0.4 to 1.5 cm, and the leaf blade can grow up to 8 cm in length and 4 cm in width, featuring a smooth and hair‐free surface. In India and Sri Lanka during the blooming period from April to May, *G*. *lactiferum* produces small, white, and pleasantly fragrant flowers arranged in clusters that resemble umbels. The corolla measures approximately 0.4–0.5 cm in diameter. As the flowering season in Sri Lanka and India approaches July and August, the plant transforms into a woody perennial climber with round‐shaped leaves, eventually giving way to fruit formation, typically between August and November (Karuppusamy, Muthuraja, and Rajasekaran 2009). Flowering has also been reported to take place from September to October (Ramzan et al. 2019). The fruit of *G*. *lactiferum* is a woody capsule that upon maturation splits open, liberating the seeds, each adorned with a tuft of silky hairs, aiding in efficient dispersal. The fruit mericarps are 6–7 cm in length, 2 cm wide, and feature a smooth, glabrous surface (IAAM 2008).

*Gymnema lactiferum* bears a striking resemblance to *G*. *sylvestre* which belongs to the same family (Wight 1834). A morphological comparison between *G*. *lactiferum* and *G*. *sylvestre* is shown in Table 2. *G*. *sylvestre*, known as Gurmar in Hindi, means “destroyer of sugar” because chewing the leaves has been observed to eliminate the sense of sweetness (Keshavamurthy and Yoganarasimhan 1990; Krishna et al. 2012). It is also referred to as Meshashringi or Madhunashini in Sanskrit, where Madhu refers to sugar and nashini means destroyer. *G*. *sylvestre* distribution extends across various countries, including India, Sri Lanka, Malaysia, Australia, Japan, Indonesia, Vietnam, tropical Africa, and some regions in China (Saneja et al. 2010).

**TABLE 2:** Comparison of *Gymnema lactiferum* and *Gymnema sylvestre*. Modified from Ramzan et al. (2019).

Character	*Gymnema lactiferum*	*Gymnema sylvestre*
Pollen—shape, and size	Subspherical shape and larger (32 μm)	Spherical shape and smaller in size (27.9 μm)
Colpi—length	1.4 μm	2.8 μm
Pollen fertility	81%	92%
Size and shape of the epidermal cells	Rectangular shaped smaller‐sized—38.9 μm	cubical to rectangular shaped large‐sized—46.7 μm
Subsidiary cells—number and shape	3 polygonal‐shaped cells	5 cubical shaped cells
Stomatal shape	Cyclocytic	Anomocytic
Stomatal aperture—size	14.8 μm	12.1 μm
Guard cells—leghth	22.5 μm	20.9 μm

The leaves of *G*. *sylvestre* contain saponins which have a sweetness‐inhibiting effect (Ye et al. 2000). Notably, research has shown that water extracts of *G*. *sylvestre* demonstrate significant decreases in glycosylated hemoglobin, blood glucose, and glycosylated plasma proteins in insulin‐dependent diabetic patients (Shanmugasundaram et al. 1990). Given its potent anti‐diabetic properties, *G*. *sylvestre* has been widely used as a treatment for diabetes since ancient times, with numerous formulations based on this plant available for managing the condition (Jarald, Joshi, and Jain 2008). LC/MS laboratory analysis has identified anti‐hyperglycemic compounds such as gymnemic acid and gymnemagenin in *G*. *sylvestre* (Kanetkar et al. 2006; Kang et al. 2012; Surveswaran et al. 2007). The plant leaves are also known to contain cardiac glycosides and anthraquinones (Patel 2017), contributing to diverse properties such as bitterness and astringency, and efficacy as an expectorant, diuretic, stomachic, liver tonic, laxative, stimulant, and antipyretic agent (Karale and Karale 2017). Overall, both *G*. *lactiferum* and *G*. *sylvestre* share similar trichome characteristics with no substantial variation (Ramzan et al. 2019), despite some other distinguishing features that set them apart.

## 3 Traditional Applications

*Gymnema lactiferum* has a rich history of traditional use, serving both as a leafy vegetable and as a remedy for various health conditions since ancient times. The milky juice or latex of the plant is known to be acrid and bitter. Interestingly, historical records repeatedly present inaccurate information about using the sap or milk for drinking or in food preparations (Chopra 1956; Lindley 1853; Loudon 1855). However, some literature has identified the above‐mentioned point as an error and claims that *G*. *lactiferum* milk is not used as a milk substitute (Tennent 1859).

Different plant parts of *G*. *lactiferum*, such as the leaves, young stems, and roots, are utilized for different purposes. In Sri Lanka, the leaves are commonly consumed either raw in salads or stir‐fried with coconut (Gunathilake, Ranaweera, and Rupasinghe 2018c). Consequently, dried leaf infusions have become integral to some Sri Lankan traditional medicinal approaches for managing diseases. For instance, “Weldehi choornaya” in Ayurveda for treating rheumatoid arthritis (Pharmacopoeia 1975; Thabrew et al. 2001) and “Mathumeha chooranam” in Siddha for treating diabetes (Thomas 2017). Moreover, it is utilized as a medicinal remedy in various traditional healing systems to address a range of health conditions, including hemorrhoids, ulcers, cancers, wounds, diabetes, parasitic worms in the stomach, and lowering cholesterol and low‐density lipoproteins (LDL) blood serum lipids.

Herbal tea made from the leaves of this plant has been used as a diuretic agent, whereas the fresh leaf pulp (prepared using water) is taken orally to help reduce constipation and intestinal ulcers. Furthermore, the plant is known to have wormicidal properties where the leaves are combined with pepper seed powder and consumed orally (Karuppusamy, Muthuraja, and Rajasekaran 2009). Native practitioners have traditionally administered chopped and boiled leaves to nursing mothers, as this is believed to enhance breast milk production for infant feeding (Tennent 1859). Even the belief in the efficacy of using this plant leaf as a dietary supplement to enhance breast milk in new mothers (Tennent 1859) persists in Sri Lanka, it is important to note that this claim lacks scientific validation. Moreover, the historical uses of this plant include as a weight‐maintenance agent, reducing the craving for sweet food. Additionally, the plant has been used in traditional medicine systems for its cooling effects and ability to rehydrate the body, and as a mild laxative agent for digestive tract comfort (Pharmacopoeia 1961).

**FIGURE 2:**
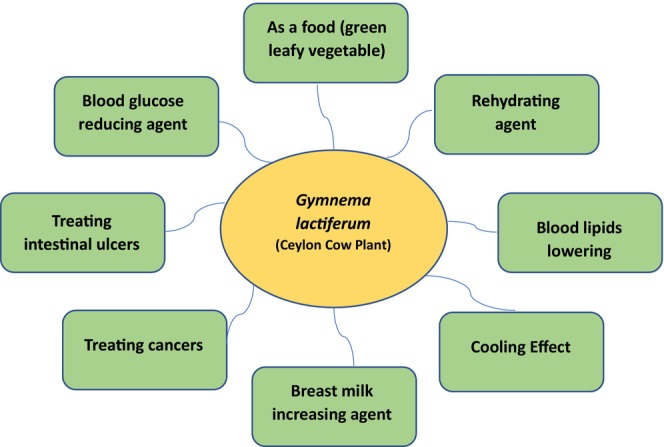
Use of *Gymnema lactiferum* in traditional medicine and its potential applications in modern research and industry.

Formulated drugs from *G*. *lactiferum* leaves are known to improve heart functions (Prabodini and Wansapala 2018). Additionally, the leaves serve as a topical treatment for recurrent boils, a condition marked by painful, pus‐filled lumps under the skin due to bacterial infection. In the realm of traditional medicine, a paste derived from crushed leaves and young stems is applied to treat boils, and both leaves and latex find use in septic wound care (Tennent 1859). Moreover, the leaves of *G*. *lactiferum* are recognized for the ability to regulate blood glucose in individuals with diabetes, a trait acknowledged since ancient times (Bandara 2008; Bandara et al. 2009; Gunathilake, Ranaweera, and Rupasinghe 2018b; Wasana et al. 2021). Clinical trials using human and animal models have provided further evidence for this claim (Bandara 2008, 2014; Bandara, Begum, et al. 2010; Bandara et al. 2009); however, the antidiabetic phytoconstituents have not been identified or isolated so far (Wasana et al. 2021). However, there are conflicting reports regarding the potential antidiabetic properties of *G*. *lactiferum*, which was defined as a non‐antidiabetic plant in a couple of reports (Perera and Handuwalage 2015; Perera and Sandaruwan 2015). Figure 2 illustrates some notable applications of *G*. *lactiferum*.

## 4 Phytochemical Constituents of *G*. *lactiferum*

### 4.1 Phenolic Compounds, Flavonoids, Carotenoids, and Minerals

Several relevant compounds, including alkaloids, flavonoids, saponins, steroids, quinones, phenolic compounds, and cardiac glycosides are present in *G*. *lactiferum* leaves (Table 3). The concentrations of alkaloids and saponins in *G*. *lactiferum* leaves were reported to be 99.1 ± 1.9 and 13.8 ± 5.3 μg/g of dry plant matter, respectively (Prabodini and Wansapala 2018). The identification and characterization of these phytochemicals in *G*. *lactiferum* leaves contribute significantly to the understanding of their therapeutic potential and may pave the way for the development of natural remedies and pharmaceutical agents.

**TABLE 3:** Reported phytochemicals and screening methods used for testing of *Gymnema lactiferum*. Modified from Prabodhani (2019).

Bioactive compound(s) tested	Test(s) used	Presence/absence in methanol extract
Phenol compounds	Lead acetate	Present
Flavonoids	Ferric chloride	
Saponins	Frothing	
Alkaloids	Mayer's Wagner's	
Steroids		
Cardiac glycosides	Keller‐Killiani	
Quinones		
Protein	Millon's	
Carbohydrates	Fehling's Bradford's	
Tannins	Ferric chloride Gelatin	Absent
Phlobatannins		
Gum and mucilage		
Leucoanthocyanidines		

The phenolic content of *G*. *lactiferum* leaves was reported as 11.03 ± 0.42 mg gallic acid equivalents (GAEs)/g dry weight (DW) in a methanolic extract, demonstrating an exceptionally high content compared to 33 other commonly used green leafy vegetables (Gunathilake and Ranaweera 2016). Additionally, this study reported that the leaves had a chlorophyll content of 23.03 ± 1.22 μg/g DW and a carotene content of 1.77 ± 0.09 mg/g DW, contributing significantly to overall nutrient density (Gunathilake and Ranaweera 2016).

Lutein is the most predominant carotenoid in *G*. *lactiferum* leaves, accompanied by the presence of β‐carotene, neoxanthin, and violaxanthin (Chandrika et al. 2010). Lutein, commonly found in green leafy vegetables, is renowned for its significant antioxidant properties. These bioactive antioxidant components may play a crucial role in preventing oxidative stress‐related diseases when *G*. *lactiferum* is incorporated into a diet. A list of bioactive compounds with reported quantitative values is presented in Table 4.

**TABLE 4:** Reported bioactive properties, mineral composition, and vitamin contents of *Gymnema lactiferum* in different studies.

Bioactive compound/mineral	Content/concentration and unit	References
Gymnemic acid	54,000 μg/g (DW)	Kathirgamanathar, Geethika, Premakumar, Balasubramaniam, et al. (2013)
Lutein	928.9 μg/g DW	Chandrika et al. (2010)
	440 μg/g DW	Gunathilake, Ranaweera, and Rupasinghe (2018a)
β‐Carotene	162.9 μg/g DW	Chandrika et al. (2010)
	320 μg/g DW	Gunathilake, Ranaweera, and Rupasinghe (2018a)
Total carotene	1770 μg/g DW	Gunathilake and Ranaweera (2016)
Violaxanthin	162.5 μg/g DW	Chandrika et al. (2010)
Neoxanthin	90.8 μg/g DW	
Total chlorophyll (a + b)	23.03 μg/g DW	Gunathilake and Ranaweera (2016)
Alkaloids	99.1 μg/g DW	Prabodini and Wansapala (2018)
Saponin	13.8 μg/g DW	
Calcium	5830 μg/g DW	
Potassium	5292.8 μg/g DW	
Sodium	2469.9 μg/g DW	
Ferric ion	146.4 μg/g DW	
	202.7 μg/g FW	Dahanayake and Ekanayake (2020)
Rutin	1470 μg/g DW	Gunathilake, Ranaweera, and Rupasinghe (2018a)
Vitamin C	1870 μg/g DW	Gunathilake, Ranaweera, and Rupasinghe (2018a)
	125.7 μg/g FW	Dahanayake and Ekanayake (2020)
Total phenolic content	11,030 ± 42 μg GAE/g DW	Gunathilake and Ranaweera (2016)
	14,520 ± 19 μg GAE/g DW	Deyalage et al. (2021)
Total flavonoid content	14,290 ± 28 μg QE/g DW	Deyalage et al. (2021)

Abbreviations: DW, dry weight; FW, fresh weight; GAE, gallic acid equivalents; QE, quercetin equivalents.

According to Prabodini and Wansapala (2018), fresh leaves from this plant contain a substantial amount of calcium and potassium. Previous research (Dahanayake and Ekanayake 2020) reports the calcium content as approximately 3.3% of dry weight (DW), showing a markedly higher amount than other commonly consumed leafy vegetables from Sri Lanka. *G*. *lactiferum* leaves have also been identified as a good source of iron and vitamin C (Dahanayake and Ekanayake 2020).

Studies underscore the potential of *G*. *lactiferum* as a source of essential minerals, although the specific form of minerals present and the amount of minerals present due to geological and plant inheritable characteristics must be noted. The bioavailability of minerals is affected by the chemical form, for example, oxalate can reduce the bioavailability of calcium if it is in the form of calcium oxalate (Noonan and Savage 1999). Microscopic observations have identified the presence of calcium oxalate rosette crystals in the petiole and stem of the closely related plant, *G*. *sylvestre* (Agnihotri et al. 2004). Moreover, plants with high calcium content, such as spinach and rhubarb, are known to have calcium in the oxalate form (Huang et al. 2020).

Phenolic compounds can be classified into soluble and insoluble forms; some are freely available whereas others are bound to other molecules (Shahidi and Yeo 2016). Gunathilake, Ranaweera, and Rupasinghe (2018b) demonstrated that *G*. *lactiferum* contains a higher concentration of free‐soluble fractions of both phenolic and flavonoid compounds compared to bound‐insoluble fractions. In this study, free soluble fractions for flavonoids and phenolic compounds were reported as 136.84 ± 3.63 rutin equivalents (RE) mmol/g DW and 408.97 ± 2.00 GAE mmol/g DW, whereas bound fractions were nearly four times lower. The distinction between free‐soluble and bound‐insoluble fractions highlights the availability and potential bioactivity of phenolic and flavonoid compounds present in *G*. *lactiferum*. According to K. Gunathilake, Ranaweera, and Rupasinghe (2018b), the increased presence of free‐soluble forms enables easier absorption from the small intestine, potentially enhancing overall bioavailability.

The yield of bioactive compounds from plant matter can be affected by various factors, such as solvent concentration, extraction time, flow rate, pH, and temperature during the extraction process (Alothman, Bhat, and Karim 2009; Robards 2003). A study to determine the optimal conditions for obtaining the highest extraction yield of bioactive compounds (total phenolics and total carotenoids) from *G*. *lactiferum* suggests that the extracting solvent concentration is a more important factor compared to time and temperature. A maximum extractable amount was obtained at an extraction temperature of 70.2°C using 19.2% aqueous ethanol for 98.2 min (Gunathilake, Ranaweera, Rupasinghe, and Rathnayaka 2018), although reporting the sample‐to‐solvent ratio would provide additional useful information, as highlighted by Tsakona, Galanakis, and Gekas (2012). Efficient techniques such as ultrasonication and microwave extraction are worthwhile to examine as these methods are known to yield higher recovery rates compared to other approaches (Azwanida 2015).

The phytochemical benefits obtained by consuming bioactive compounds depend on bioavailability and bioaccessibility after intake and subsequent metabolism (Tagliazucchi et al. 2010). Before exhibiting any physiological effects, these compounds need to retain bioactivity during the digestive processes before the point of optimal absorption in the digestive tract. In the case of *G*. *lactiferum*, the total polyphenolic and carotenoid contents are significantly affected (Gunathilake, Ranaweera, and Rupasinghe 2018b) during human digestion compared to the original amount present in the plant material. The bioavailability of lutein, for instance, was found to be 39.2% of the original methanolic extract, whereas the bioavailability of β‐carotene was relatively lower. Interestingly, this gastrointestinal simulation study further revealed that rutin, a common flavonoid found in green leaves, showed much higher content in the intestinal media compared to that present in the native leaves. Since rutin and beta carotene are not bioaccessible after digestion but still prevail in high amounts in gastrointestinal media (Gunathilake, Ranaweera, and Rupasinghe 2018b). The observed increase in rutin in the intestinal media is somewhat surprising and could be attributed to the effect of specific enzymes prevailing in the digestive media and the presence of bacteria. Gut microbiota may metabolize complex flavonoid structures into simpler forms (Braune and Blaut 2016). The longer time inside the digestive tract, nearly three hours compared to the half‐hour used for methanolic extraction, may contribute to this disparity. However, such a comparison is not valid due to different methods, solvents, and time.

Deyalage et al. (2021) investigated the impact of commonly used cooking temperatures (70°C, 120°C, and 170°C) on total phenolic content (TPC) and total flavonoid content (TFC) of *G*. *lactiferum*. A consistent and gradual increase in TPC and TFC with rising temperature was observed, reaching levels 140% and 200% higher than at 70°C, respectively. Furthermore, both DPPH and FRAP assays showed greater inhibition at 170°C. Deep frying at 170°C for 5 min had the highest antioxidant activity, offering practical applications for cooking to maximize beneficial compound retention. TPC and TFC values of *G*. *lactiferum* under different temperature treatments used for cooking in that study are presented in Table 5.

**TABLE 5:** Total polyphenol content and total flavonoid content of *Gymnema lactiferum* under different temperature treatments used for cooking. Modified from Deyalage et al. (2021).

Bioactive compound	Original sample content	At 70°C	At 120°C	At 170°C
Total polyphenol content (mg GAE/g DW)	14:52 ± 0:19^f^	15:34 ± 0:08^e^	17:23 ± 0:20^d^	20:28 ± 0:12^c^
Total flavonoid content (mg QE/g DW)	14:29 ± 0:28^d^	9:55 ± 0:12^f^	22:60 ± 1:01^b^	28:84 ± 0:62^a^

*Note:* Values with different superscript letters within the same row are significantly different (*p* > 0.05). Values are presented as mean ± SD, *n* = 3.

Abbreviations: DW, dry weight; GAE, gallic acid equivalents; QE, quercetin equivalents.

This effect of cooking temperature aligns with results from other plants. Domestic cooking methods (conventional, pressure, and microwave) induced higher TPC in nine out of 11 plants studied, showing increases ranging from 111% to 211%, and coriander (*Coriandrum sativum*) leaves exhibited the most significant rise in phenolic content (Sreeramulu et al. 2013). The observed increase in phenolic content can be explained by the likelihood of macromolecular structures breaking down under elevated cooking temperatures. This breakdown may result in the production of simpler polyphenols, including soluble forms, which may react more readily with the test reagents (Bunea et al. 2008).

The diversity in phenolic compounds and their binding to cell structures contributes to variations in the hydrolysis of phenolic bonds under specific heating conditions and with different food types (Faller and Fialho 2009). Heat treatments may deactivate polyphenol oxidase which typically prevents unwanted oxidation and polymerization reactions in polyphenols (Hiemori, Koh, and Mitchell 2009). This enzymatic inactivation thus may help to preserve phytochemicals within a food product.

### 4.2 Gymnemic Acid

*Gymnema lactiferum* contains 100–270 mg of gymnemic acid per 5 g of dried leaves, depending on the solvent used for extraction. Among the different solvents tested, 50% and 70% ethanolic extracts were identified as the two most effective extracting media for obtaining higher yields (Kathirgamanathar, Geethika, Premakumar, Balasubramaniam, et al. 2013). Methanol reportedly yields higher recovery rates of gymnemic acids from *G*. *sylvestre* (Krishna et al. 2012).

**FIGURE 3:**
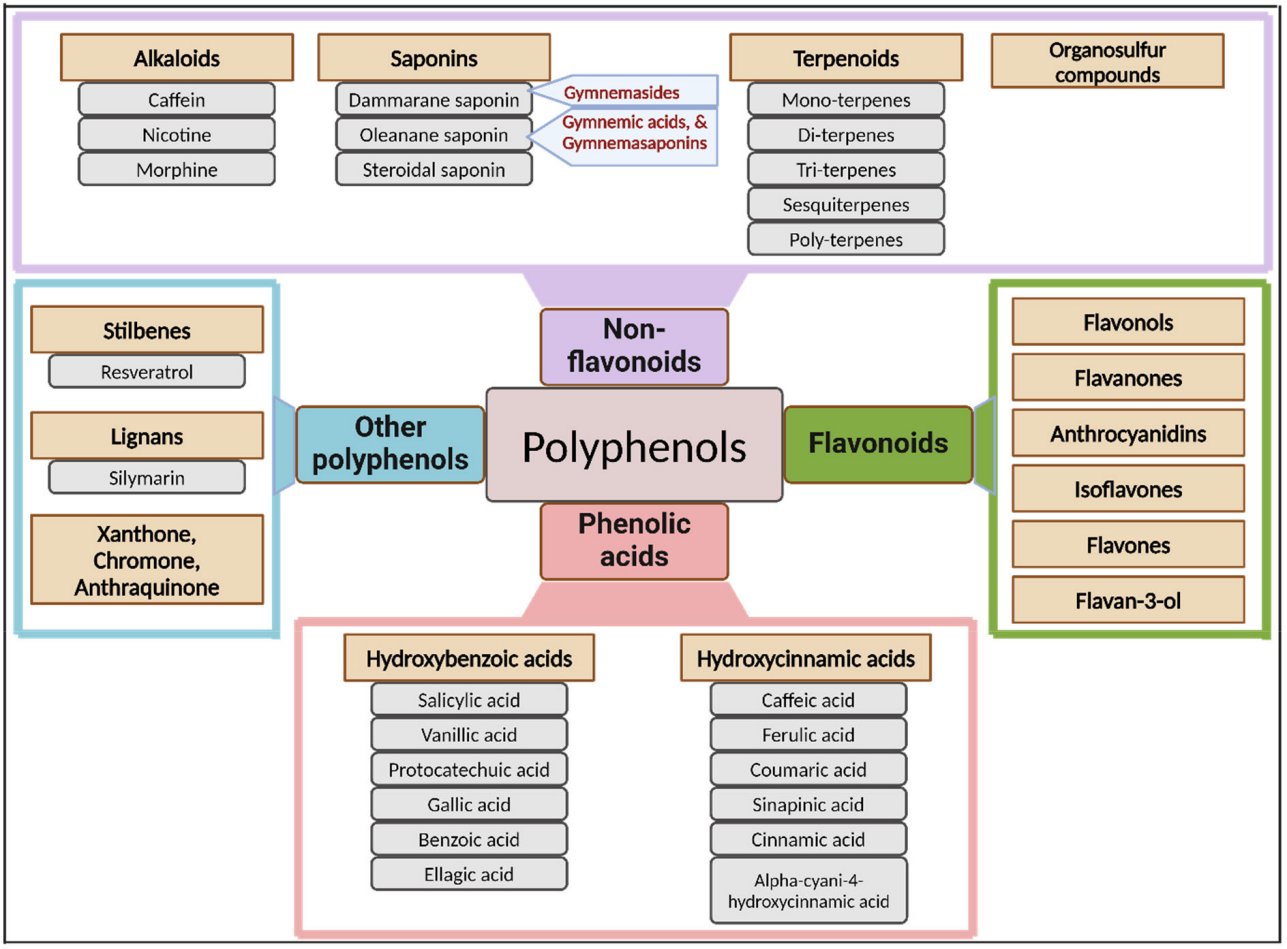
Polyphenol classification and the corresponding position of gymnemic acid.

Gymnemic acids have reported anti‐diabetic and anti‐inflammatory properties (Krishna et al. 2012; Liu, Kiuchi, and Tsuda 1992) and have played a pivotal role in diabetes management in traditional medicine for generations (Kathirgamanathar, Geethika, Premakumar, Balasubramaniam, et al. 2013; Kurihara 1969). These compounds are distributed throughout the plant, with the highest concentration found in shoot tips and the lowest in seeds (Tiwari, Mishra, and Sangwan 2014). A significant milestone was achieved in 1889 when Hooper's research discovered potassium salts of gymnemic acid (Hooper (1889) as cited in Stoecklin (1969)). Gymnemic acids are categorized as triterpene glycosides (Mander and Liu 2010), primarily falling under the oleanane and dammarane classes (Tiwari, Mishra, and Sangwan 2014). Figure 3 illustrates the polyphenol classification, showing the position of gymnemic acid. A significant subgroup encompasses nine closely related acidic glycosides, prominently referred to as gymnemic acids A–D. Additionally, multiple constituents are found within the class of gymnemic acids, denoted as gymnemic acids I–VII, gymnemosides A–F, and gymnemasaponins (Tiwari, Mishra, and Sangwan 2014).

**FIGURE 4:**
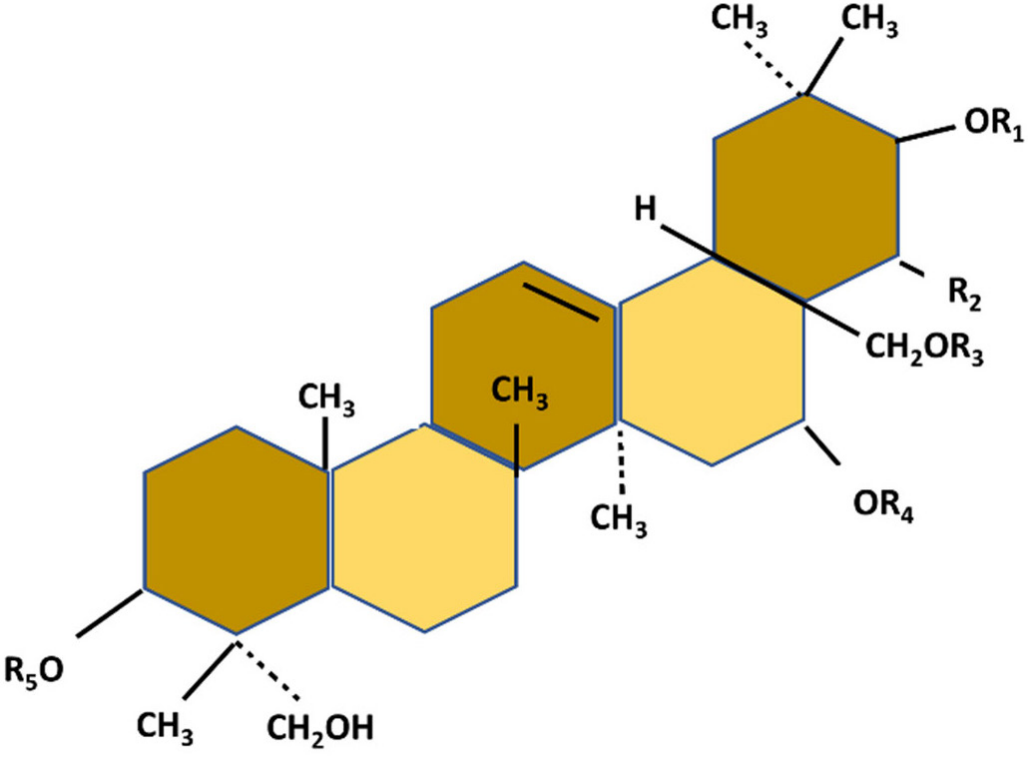
Fundamental chemical structure of gymnemic acid.

Distinct variations of gymnemic acid, characterized by diverse acyl groups, have been extracted from *G*. *sylvestre* leaves, with chemical structures identified. The removal of the acyl group results in reduced anti‐sweetness activity (Mander and Liu 2010). At the heart of gymnemic acids lies the aglycone gymnemagenin, with the chemical formula C_30_H_50_O_6_ (see Figure 4). This foundational structure is augmented with a sugar group, often glucuronic acid, and various ester groups, contributing to the formation of distinct gymnemic acids. The molecular diversity stemming from sugar and ester variations underscores the multifaceted nature of gymnemic acids and the potential for diverse biological activities (Sheng and Sun 2011).

The antidiabetic properties of gymnemic acids arise from a mechanism that slows down the absorption of glucose from the intestine into the bloodstream (Patel, Gadewar, and Tripathi 2012). Gymnemic acid molecules bear a resemblance to glucose molecules, enabling them to block the receptor sites on taste buds, thereby reducing the ability to taste sweetness and making sweet food less attractive. Similarly, these molecules bind to receptor sites in the absorptive outer layers of the intestine, blocking the absorption of glucose molecules and leading to lower blood glucose concentrations after a meal (Vaidya 2011). Furthermore, gymnemic acids enhance the activity of enzymes responsible for utilizing glucose through insulin‐dependent pathways, increase phosphorylase activity, decrease gluconeogenic enzymes and sorbitol dehydrogenase, and inhibit sorbitol dehydrogenase. These are just a few of the potential mechanisms by which gymnemic acids exert anti‐hyperglycemic effects (Agarwal et al. 2000).

## 5 Biological Activity of *G*. *lactiferum*

### 5.1 Antioxidative Activity

The concentration of antioxidants present in plant‐based ingredients depends upon post‐harvest processing steps. Short‐term frying and boiling have been found to enhance antioxidant capacity and increase the bioaccessibility of bioactive constituents (Hossain et al. 2017). Deyalage et al. (2021) investigated the impact of domestic thermal cooking treatments on *G*. *lactiferum* leaves and found a positive effect on antioxidant capacity and polyphenolic concentration compared to the uncooked state, suggesting a positive correlation between heat treatment intensity and antioxidant activity. In that study, *G*. *lactiferum* showed the lowest 2,2‐diphenyl‐1‐picrylhydrazyl (DPPH) and ferric‐reducing antioxidant power (FRAP) scavenging ability among the three tested plants in an uncooked state but did not align with the positive correlation between antioxidant capacity and polyphenolic content reported by Gunathilake and Ranaweera (2016). This apparent discrepancy may arise from limitations associated with the DPPH assay method in identifying some phenolic structures and the lower responsiveness of the DPPH radical to some antioxidants present in the media (Gregoris et al. 2013). Deyalage et al. (2021) did not explore the potential impact of extended cooking times at lower temperatures. Although frying for 5 min was reported as the optimal cooking method for preserving bioactivities, this is not recognized as beneficial to preserving the anti‐inflammatory properties of *G*. *lactiferum* compared to boiling or steaming (Gunathilake, Ranaweera, and Rupasinghe 2018e).

A methanolic extract from *G*. *lactiferum* was tested for total phenolic, carotene, and chlorophyll content, and for total antioxidant capacity, lipid peroxidation, reducing power, and DPPH radical scavenging. A positive correlation was observed between antioxidant assays and polyphenolic content, highlighting the significance of polyphenols for inducing antioxidant activity (Gunathilake and Ranaweera 2016). The values obtained from other antioxidant assays are presented in Table 6. These findings highlight the potent antioxidant potential of *G*. *lactiferum* compared to other plants examined. However, this contradicts the results obtained for 2,2′‐azino‐bis(3‐ethylbenzothiazoline‐6‐sulfonic acid) (ABTS) of 1.03 ± 0.13 mM Trolox equivalents (TE)/g and DPPH of 0.11 TE/g DW radical scavenging activities, where the methanolic extraction, used as the control in the study, exhibited the least activity among the tested varieties of the three plants (Deyalage et al. 2021). These results may be attributed to variations in the concentration of methanol used and differences in the drying methods employed.

**TABLE 6:** 2,2‐Diphenyl‐1‐picrylhydrazyl (DPPH) radical scavenging activity, reducing power, lipid peroxidation activity, and total antioxidant capacity of *Gymnema lactiferum*. Modified from K. P. P. Gunathilake and Ranaweera (2016).

Antioxidant analyzing method	Value
DPPH radical scavenging activity (%inhibition)	14.22 ± 0.11
Reducing power assay (mg of AAE/g DW)	4.47 ± 0.07
%Inhibition of lipid peroxidation	73.92 ± 10.41
Total antioxidants capacity (mg of AAE/g DW)	12.62 ± 0.47

Abbreviations: AAE, ascorbic acid equivalents; DPPH, 2,2‐diphenyl‐1‐picrylhydrazyl; DW, dry weight.

In vitro digestion of *G*. *lactiferum* showed a progressive extraction of polyphenols, flavonoids, and carotenoids from the food matrix (Gunathilake, Ranaweera, and Rupasinghe 2018b); however, the amount of dialyzable soluble antioxidants available for final uptake after digestion was considerably lower than the initial antioxidant content and activity present in the original leaf sample. Antioxidant activity values at different points during digestion using different assays are presented in Table 7. These findings highlight the importance of understanding the impact of the food matrix on the bioavailability and bioaccessibility of antioxidants and other bioactive compounds after consumption. In vitro digestion models simplify the processes occurring in the human digestive system, and may not be consistent with the results from colonial catabolism and in the presence of gut‐specific microbes (Gunathilake, Ranaweera, and Rupasinghe 2018b; Mosele et al. 2016). Methods using different cell lines and animal in vivo trials in a more complex biological context are necessary to validate in vitro digestion results.

**TABLE 7:** Antioxidant activity of *Gymnema lactiferum* samples at various stages of digestion using different assays. Modified from Gunathilake, Ranaweera, and Rupasinghe (2018b).

Phase	ABTS radical scavenging ability (mmol AAE/g leaves (FW))	Singlet oxygen radical scavenging ability (mmol GAE/g leaves (FW))	Total antioxidant capacity (mmol AAE/g leaves (FW))
Original content in methanol extract	37.3 ± 4.9	120.8 ± 13.0	153.4 ± 17.7
Gastric	70.8 ± 2.6	168.2 ± 22.2	137.4 ± 10.4
Gastro‐intestinal	276.9 ± 5.0	191.7 ± 9.1	86.9 ± 6.0
Dialysis	50.9 ± 0.4	21.6 ± 2.1	26.6 ± 1.1

Abbreviations: AAE, ascorbic acid equivalents; ABTS, 2,2′‐azino‐bis(3‐ethylbenzothiazoline‐6‐sulfonic acid); FW, fresh weight; GAE, gallic acid equivalents.

Ensuring the effectiveness of bioactive compounds in functional foods requires consideration of bioavailability and bioaccessibility. Encapsulation can protect bioactive compounds from degradation before absorption in the body (Zabot et al. 2022) and it may emerge as a useful technique for the delivery of bioactive components derived from *G*. *lactiferum*.

### 5.2 Anti‐Hyperglycemic Activity

Diabetes has become an increasingly common disorder affecting a substantial percentage of the global population since the early 1800s (Vaidya 2011), ranking as the ninth‐leading cause of death (Shin, Connolly, and Kabytaev 2023). Medicinal plants have long been known to contain various bioactive compounds, such as polyphenols, flavonoids, terpenoids, and coumarins, which are recognized for their ability to lower blood glucose. Results from a human clinical trial showed significant effects on fasting blood glucose, a postprandial glucose‐lowering effect, and a substantial reduction in glycated hemoglobin (HbA1C) concentrations, measuring the average blood glucose concentration throughout for 3 months (Bandara et al. 2009). This study did not report a significant reduction in fasting blood glucose in normoglycemic healthy rats over a similar period, nor did it show a significant effect on blood glucose concentrations after the glucose challenge of those healthy rats (Bandara, Ekanayake, et al. 2010). Another study on type 2 diabetic‐induced rats reported a significant reduction in fasting blood glucose (Bandara, Begum, et al. 2010). These reports suggest that *G*. *lactiferum* leaf powder possesses anti‐hyperglycemic properties that may stem from mechanisms such as stimulation of insulin secretion, augmentation of insulin activity, or inhibition of glucose absorption. We cannot conclude that *G*. *lactiferum* leaf powder can be effectively used as a functional food for individuals with type 2 diabetes due to the small sample size in these two studies, the relatively short intervention time, and the problem of extrapolating the results of rat trials to humans.

Protein glycation, a pivotal factor in chronic diabetic complications, underscores the importance of identifying medicinal plants with inhibitory properties. In a comprehensive study evaluating 10 plants, *G*. *lactiferum* showed the lowest inhibition of protein glycation, emphasizing the complexity of the role of *G*. *lactiferum* in managing complications from chronic diabetes. In the context of postprandial hyperglycemia, a critical aspect of diabetes management, two studies revealed no significant inhibitory effects on α‐amylase and α‐glucosidase from *G*. *lactiferum* or the related plant, *G*. *sylvestre* (Poongunran et al. 2014, 2015). These findings challenge the notion of the effectiveness of *G*. *lactiferum* in targeting these therapeutic enzymes associated with glucose metabolism. However, similar results (i.e., lower α‐amylase and α‐glucosidase activities) were observed for *G*. *sylvestre*, a closely related and well‐known antidiabetic plant in the above‐mentioned study. The anti‐hyperglycemic effects of these plants might be due to a different mechanism than the ones investigated in those studies.

### 5.3 Cholesterol Regulating Activity

High blood cholesterol continues to be a significant health concern that adversely affects quality of life. Elevated blood cholesterol concentrations have been linked to cardiovascular conditions, including heart disease and stroke (Glanz 1988). Dyslipidemia, characterized by increased concentrations of LDL or “bad cholesterol” and elevated triglycerides, along with decreased concentrations of high‐density lipoproteins (HDL) or “good cholesterol”, has been implicated in the development of hyperglycemia or high blood glucose concentrations levels. Understanding the complex interactions between lipid metabolism and glucose homeostasis is essential for effectively managing and preventing hyperglycemia. This relationship is evident in the simultaneous occurrence of hypercholesterolemia and hypertriglyceridemia in diabetic‐induced rats (Sharma, Dwivedi, and Swarup 1997).

The beneficial effects of a *G*. *lactiferum* leaf suspension in lowering serum lipid concentrations have been observed in diabetic patients with hypercholesterolemia, leading to a significant decline in serum cholesterol and LDL (Bandara et al. 2009). A decline in serum cholesterol concentrations was also reported in diabetic‐induced rats, but any significant changes were not observed in serum HDL and triglycerides (Bandara, Begum, et al. 2010). No significant effect was reported for serum alanine transferase and creatinine concentrations among diabetic patients (Bandara et al. 2009), suggesting that the treatment did not have detrimental effects on the liver and kidneys of the patients.

Despite the positive clinical results of *G*. *lactiferum* in reducing blood cholesterol, the mechanisms behind this effect remain unknown. This hypocholesterolemic effect could be due to an indirect impact of reducing blood glucose concentrations, or the presence of active compounds that inhibit cholesterol synthesis or enhance the excretion of cholesterol or bile salts. Indeed, the limitations of small sample sizes in both animal and human trials coupled with the short treatment duration hinder our ability to draw definitive conclusions. Uncovering mechanisms will potentially lead to therapeutic applications and the development of interventions to lower cholesterol.

### 5.4 Anti‐Inflammatory Activity

Inflammation is a crucial component of the general immune response, activated in response to physical harm or injury (Ferrero‐Miliani et al. 2007). This complex process involves aspects such as increased vascular permeability, changes in membrane properties, and protein denaturation. In certain situations, inflammation induces dilation of blood capillaries and enhances capillary permeability, facilitating an increased blood supply to the affected area (Chandra et al. 2012).

*Gymnema lactiferum* was reported to have anti‐inflammatory properties attributed to the polyphenol content and antioxidant properties (Gunathilake, Ranaweera, and Rupasinghe 2018d). This study reported a relatively high inhibition of lipoxygenase activity and a significant correlation between anti‐inflammatory properties and the level of total flavonoids, polyphenols, and carotenoids. Polyphenols have been found to impede the cascade processes of arachidonic acid metabolism by inhibiting lipoxygenase activity (Ammon et al. 1992; Trouillas et al. 2003). The inhibition of protein denaturation, lipoxygenase activity, and hemolysis are known as anti‐inflammatory properties (Gunathilake, Ranaweera, and Rupasinghe 2018d). These properties may vary based on cooking methods and leaf types. Lipoxygenase inhibiting activity was reported as lower (*p* < 0.05) in cooked *G*. *lactiferum* leaves when compared to the native amount in the extract. Protein denaturation is a well‐documented factor related to inflammatory reactions and since the membrane of red blood cells is quite similar to the lysosomal cell membrane, red blood cell hemolysis inhibition provides an understanding of the process of inflammation (Umapathy et al. 2010). Steaming of *G*. *lactiferum* leaves resulted in 44.8% inhibition of protein denaturation and was shown to significantly lower (*p* < 0.05) activity in boiling, whereas frying had the lowest activity (Gunathilake, Ranaweera, and Rupasinghe 2018e). Furthermore, the anti‐inflammatory activities were found to vary, depending on the different digestion fractions throughout the digestive tract under in vitro digestive conditions (Gunathilake, Ranaweera, and Rupasinghe 2018c). Lipoxygenase inhibitory activity was consistently low in this study, measuring < 10%, across all digestion fractions throughout the digestive tract, including simulated gastric, intestinal, and dialyses phases. However, a significantly higher hemolysis inhibitory activity (nearly more than 14%, *p* < 0.05) was reported in the gastric phase than in the intestinal and dialysis fractions, whereas a higher proteinase inhibitory activity (nearly more than 29%) in the intestinal phase, compared to the gastric and dialysis fractions, was reported.

## 6 Future Directions

We suggest the following key recommendations to explore and utilize the medicinal potential of *G*. *lactiferum*.

1. Microencapsulation may be used to enhance the stability and bioavailability of *G*. *lactiferum* extracts. This approach could facilitate fortification into a broader range of functional food products, which in turn, expand consumer accessibility.
2. A comprehensive nutrient (especially minerals) analysis of *G*. *lactiferum* leaves should be carried out, including the form and bioavailability of calcium. The characteristics of *G*. *lactiferum* sap (milk) may uncover potential therapeutic applications.
3. Rigorous safety and toxicological assessments should be undertaken to evaluate potential harmful effects and establish exposure limits for different extracts from *G*. *lactiferum*. Such work should also delve into phytochemical aspects at the molecular level to elucidate mechanisms of action through cellular signaling pathways.
4. Identification of additional potential bioactive compounds present in *G*. *lactiferum*
5. Screening of different solvents and extraction methods for increasing activity and yield of bioactive compounds from *G*. *lactiferum*.
6. Animal cell line studies will be a more straightforward and desirable option to evaluate the impact on diabetes and provide a more comprehensive understanding of the mechanism for the anti‐hyperglycemic activity of *G*. *lactiferum*. Due to the presence of more relevant physiological, morphological, and biochemical aspects, including receptors, this will have the potential to elucidate the specific mechanisms responsible for this activity.
7. Utilizing plant tissue cultures and in vitro propagation techniques can identify alternative pathways in synthesizing critical secondary metabolites in large‐scale commercial applications, instead of using plant extracts. Uncovering this aspect in *G*. *lactiferum* will provide insights into advancements in plant‐based pharmaceuticals and natural product chemistry in the future.

## 7 Conclusions

Rich in bioactive compounds, including gymnemic acid and essential carotenoids such as lutein and β‐carotene, *G*. *lactiferum* stands as a valuable resource for the development of functional food products with enhanced health‐promoting attributes. Functional health attributes, such as anti‐hyperglycemic properties and regulation of blood cholesterol, have been identified in a clinical study. The antioxidative effects and anti‐inflammatory characteristics have also been identified in some of the laboratory experiments. Nonetheless, the mechanisms of action for such health‐promoting properties remain largely unexplored, meaning that future work will be needed to confirm and validate the reported claims further. Larger sample sizes, coupled with human clinical trials, model cell culture studies, and in vivo animal models, will contribute to a more reliable and scientifically robust understanding of the health properties of *G*. *lactiferum*, which stands as a promising candidate for future advancements in both functional food and medicine. Lastly, most of the corresponding chemical profiles associated with such properties are yet to be identified. Specifically, the use of novel techniques for extraction, compound quantification, identification, and encapsulation like totally new aspects will be a great delivery method of the bioactivities in the extract, which can be a positive contribution to the food sector in the future.

## Author Contributions

**D. M. K. P. Weerasinghe:** conceptualization (equal), data curation (lead), formal analysis (lead), investigation (lead), methodology (lead), project administration (lead), resources (equal), software (equal), visualization (equal), writing – original draft (lead). **L. Brough:** data curation (equal), methodology (equal), project administration (equal), resources (equal), supervision (equal), validation (equal), visualization (equal), writing – review and editing (equal). **D. W. Everett:** data curation (equal), methodology (equal), project administration (equal), resources (equal), supervision (equal), validation (equal), visualization (equal), writing – review and editing (equal). **A. Rashidinejad:** conceptualization (lead), data curation (equal), formal analysis (equal), funding acquisition (equal), investigation (equal), methodology (equal), project administration (equal), resources (lead), supervision (lead), validation (lead), visualization (equal), writing – review and editing (lead).

## Conflicts of Interest

The authors declare no conflicts of interest.

## Data Availability

Data will be available from the corresponding author upon request.
